# Lysophosphatidic Acid Disrupts Junctional Integrity and Epithelial Cohesion in Ovarian Cancer Cells

**DOI:** 10.1155/2012/501492

**Published:** 2012-04-22

**Authors:** Yueying Liu, Rebecca Burkhalter, Jaime Symowicz, Kim Chaffin, Shawn Ellerbroek, M. Sharon Stack

**Affiliations:** ^1^Department of Chemistry and Biochemistry, University of Notre Dame, 1234 Notre Dame Avenue, A200D Harper Hall, Notre Dame, IN 46557, USA; ^2^Harper Cancer Research Institute, University of Notre Dame, 1234 Notre Dame Avenue, A200D Harper Hall, Notre Dame, IN 46557, USA; ^3^Department of Medical Pharmacology and Physiology, University of Missouri School of Medicine, Columbia, MO 65212, USA; ^4^Department of Cell and Molecular Biology, Northwestern University, Chicago, IL 60611, USA; ^5^Department of Chemistry, Wartburg College, Waverly, IA 50677, USA

## Abstract

Ovarian cancer metastasizes via exfoliation of free-floating cells and multicellular aggregates from the primary tumor to the peritoneal cavity. A key event in EOC metastasis is disruption of cell-cell contacts via modulation of intercellular junctional components including cadherins. Ascites is rich in lysophosphatidic acid (LPA), a bioactive lipid that may promote early events in ovarian cancer dissemination. The objective of this paper was to assess the effect of LPA on E-cadherin junctional integrity. We report a loss of junctional E-cadherin in OVCAR3, OVCA429, and OVCA433 cells exposed to LPA. LPA-induced loss of E-cadherin was concentration and time dependent. LPA increased MMP-9 expression and promoted MMP-9-catalyzed E-cadherin ectodomain shedding. Blocking LPA receptor signaling inhibited MMP-9 expression and restored junctional E-cadherin staining. LPA-treated cells demonstrated a significant decrease in epithelial cohesion. Together these data support a model wherein LPA induces MMP-9 expression and MMP-9-catalyzed E-cadherin ectodomain shedding, resulting in loss of E-cadherin junctional integrity and epithelial cohesion, facilitating metastatic dissemination of ovarian cancer cells.

## 1. Introduction

Epithelial ovarian cancer (EOC) is the leading cause of death from gynecologic malignancy in the United States. In 2010, approximately 21,880 women were newly diagnosed with EOC and 13,850 women died from complications due to disseminated intraperitoneal metastasis [1]. Clinically, tumors often involve the ovary and omentum, with diffuse, multifocal intraperitoneal metastases and malignant ascites. As 75% of women with EOC are initially diagnosed with previously disseminated intra-abdominal disease, a more detailed understanding of factors that promote successful metastasis can ultimately improve patient survival.

In women with advanced EOC, obstruction of peritoneal lymphatics together with enhanced vascular permeability results in accumulation of malignant ascites, and the presence of ascites is an adverse prognostic factor [1–4]. Ascites is comprised of >200 proteins, tumor and inflammatory cells, and cytokines. A major bioactive component of EOC ascites is the lipid lysophosphatidic acid (LPA). Elevated LPA levels (up to >80 *μ*M) are detectable in 98% of patients with EOC, including 90% of patients with stage I disease [5–10]. Multiple studies have shown that LPA contributes to tumor development, progression, and metastasis through binding to a subfamily of G protein-coupled receptors termed LPA receptors (LPAR), thereby effecting expression of proteases and growth factors and modulating migration of a variety of cells [11–19]. In EOC, treatment with LPA *in vitro* results in an enhanced metastatic phenotype, characterized by increased proteolytic activity, stimulation of motility, and more aggressive invasive behavior [11, 12, 20, 21]. LPA also enhances adherens junction dissolution and colony dispersal and promotes epithelial-mesenchymal transition [13, 22]. Primary differentiated EOC display abundant expression of the cell-cell junctional protein E-cadherin; however, reduced E-cadherin staining is found in late-stage carcinomas and data suggest that loss of E-cadherin expression or function is a factor in EOC progression from well-differentiated lesions to poorly differentiated tumors and metastases [2–4]. As matrix metalloproteinases (MMPs) are implicated in E-cadherin ectodomain shedding [23–26] and LPA is linked to altered MMP expression [12–14], the current study was designed to evaluate a potential functional link between LPA, posttranslational regulation of E-cadherin function, and epithelial cohesion in EOC.

## 2. Materials and Methods

### 2.1. Cell Culture

OVCA429 and OVCA433 cells, generously provided by Dr. Robert Bast (M. D. Anderson Cancer Center, Houston, TX), were maintained in MEM, 10% fetal bovine serum, penicillin/streptomycin, amphotericin B, nonessential amino acids, and sodium pyruvate at 37°C in 5% CO_2_. OVCAR-3 cells were maintained in RPMI 1640 (American Type Culture Collection, Manassas, VA), 20% fetal bovine serum, penicillin/streptomycin, amphotericin B, nonessential amino acids, sodium pyruvate, and insulin from bovine pancreas (10 mg/L) at 37°C in 5% CO_2_.

### 2.2. Materials

Lyophilized LPA was purchased from Cayman Chemical (Ann Arbor, MI) and reconstituted in dH_2_O at 2 mM. LPA receptor inhibitor (LPARI), Ki16425, was purchased from Cayman Chemical (Ann Arbor, MI). The *K*_*i*_ of the inhibitor for LPA_1–3_ receptors is 0.3–6 *μ*M, with a working concentration of 10 *μ*M sufficient to block LPAR function [27]. The protease inhibitors uPA Stop and GM6001 were purchased from American Diagnostica Inc. (Stamford, CT) and Chemicon (Temecula, CA), respectively. Function-blocking anti-MMP-9 antibody was purchased from Calbiochem (Darmstadt, Germany), and the E-cadherin antibody hecd-1 was purchased from Calbiochem, Darmstadt, Germany or Zymed, San Francisco, CA.

### 2.3. Immunoprecipitation and Western Blot Analysis

Cells were subcultured in six-well plates to 70% confluence. After 1 day, cells were serum starved overnight in the appropriate medium. In some experiments, cells were pretreated with inhibitor (or equivalent concentrations of DMSO) for 1.5 to 3 hours in serum-free media. Cells were then treated with LPA for 18–24 hours before lysis in modified radioimmunoprecipitation assay lysis buffer (mRIPA; 50 mmol/L Tris pH 7.5, 150 mmol/L NaCl, 0.1% SDS, 1% Triton X-100, 5 mmol/L EDTA). Protein concentrations of the resulting lysates were determined using the Bio-Rad (Hercules, CA) protein assay. Lysates (ranging from 30 to 70 *μ*g, as indicated) were electrophoresed on an 8% SDS-polyacrylamide gel, electroblotted to a polyvinylidene difluoride (PVDF) membrane [28], and blocked in 5% milk/TBS-T (25 mmol/L Tris pH 7.5, 150 mmol/L NaCl, 0.1% Tween 20) or 3% bovine serum albumin/TBS-T at room temperature for 1 to 3 hours. Blots were incubated overnight with 1 : 1,000 dilution of the primary antibody. The immunoreactive bands were visualized using peroxidase-conjugated anti- mouse or rabbit immunoglobulin G (1 : 5,000 in 3% bovine serum albumin/TBS-T) and enhanced chemiluminescence. To evaluate loading controls, blots were stripped of primary antibody using a low-pH buffer (400 mmol/L glycine pH 2.5), blocked again in 3% bovine serum albumin/TBS-T, and reprobed with primary antibody. Western bands were quantified by densitometric quantitation. Results were normalized against the densitometric reading for untreated cells.

### 2.4. Gelatin Zymography

Serum-free conditioned medium was resolved using gelatin-containing SDS-polyacrylamide gels, followed by washing with 2.5% Triton X100 and incubation in zymography buffer (20 mM glycine/10 mM calcium chloride/1 *μ*M zinc chloride) at 37 degrees Celsius for 24–48 hours. Gels were stained with Coomassie blue (45% methanol, 3 g/L Coomassie Brilliant Blue, and 10% acetic acid in dH_2_O), and destained in acetic acid solution (10% acetic acid and 15% methanol in dH_2_O). Under serum-free conditions, MMP-9 is detected in the proenzyme form [28].

### 2.5. Immunofluorescence Microscopy

Cells were subcultured on glass coverslips in six-well plates to 80% confluence. Following overnight serum-starvation in appropriate medium, cells were treated with LPA (1–80 *μ*M) for 18–24 hours. In some experiments, cells were pretreated prior to addition of LPA with inhibitor (or equivalent concentrations of DMSO) for 1.5 to 3 hours (GM6001-MMP inhibitor, Chemicon, 25 *μ*M; Ki16425-LPA receptor inhibitor, Cayman Chemical, 40 *μ*M; uPA Stop-uPA inhibitor, American Diagnostica, 2.5 *μ*M). Cells were fixed in 4% PFA for 20 minutes at room temperature, followed by immunostaining with anti-E-cadherin (hecd-1 clone, Zymed, San Francisco, CA; 1 : 300) and Alexa-Fluor 488-conjugated secondary antibody (1 : 500). Fluorescence microscopy was performed using an Olympus IX-81 spinning disc confocal microscope. Semi-quantitative analysis of E-cadherin junctional integrity was determined by counting a minimum of 12 fields per treatment (at least 100 cells overall) and scoring as positive the number of cells with two remaining fluorescent cell-cell borders.

### 2.6. Dispase-Based Dissociation Assay

To monitor relative changes in epithelial cohesion, dispase-based dissociation assays were performed as previously described [29]. Briefly, cells were subcultured (triplicate per condition) in 60-millimeter dishes to 80% confluence in MEM. Cultures were washed twice in PBS before being incubated with 2 mL dispase in DMEM/F12 (Stem Cell Technologies, Vancouver, British Columbia, Canada) until the cell-cell cohesive monolayer detached from the culture plate. Subsequently, the detached cell monolayer was washed in PBS, transferred to a 15 mL conical tube, and subjected to 50 inversion cycles on a bench-top rocker. All cell fragments were placed into tissue culture dishes and fragment number enumerated by light microscopy. The assay was repeated twice and analyzed statistically by Student's *t*-test.

## 3. Results

**Figure 1:**
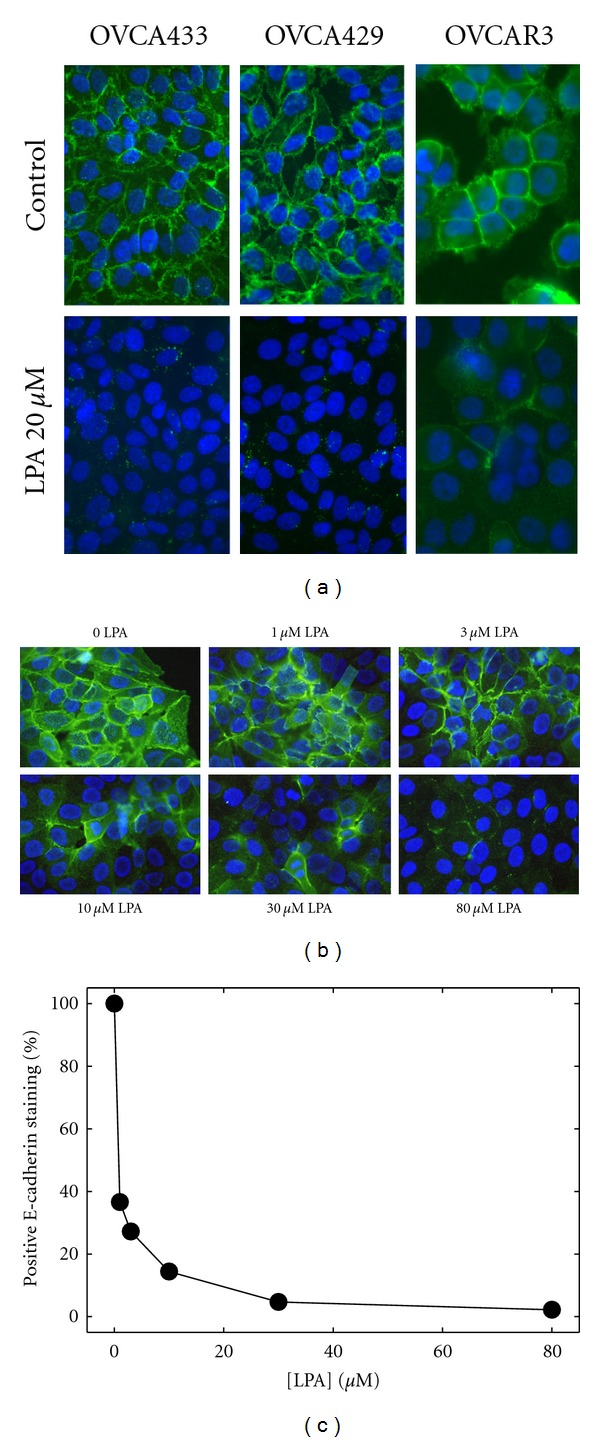
LPA induces E-cadherin junction disruption in EOC cells. (a) Confluent monolayers of OVCA433, OVCA429, or OVCAR3 cells, as indicated, were treated with LPA (20 *μ*M) for 18–24 hours and processed for immunofluorescence staining for E-cadherin using anti-E-cadherin ectodomain antibody (1 : 300) and Alexa Fluor 488-conjugated secondary antibody (1 : 500; green). Blue-DAPI-stained nuclei. (b) To evaluate the dose-dependence of LPA-induced junction loss, OVCA429 cells were treated with LPA at the concentrations indicated for 24 hours and processed for E-cadherin immunofluorescence (green) as described in (a) above. Blue-DAPI-stained nuclei. (c) Junction loss was quantified by counting the number of cells/field with two remaining E-cadherin immunostained borders in a minimum of 5 fields per treatment (at least 100 cells).

**Figure 2:**
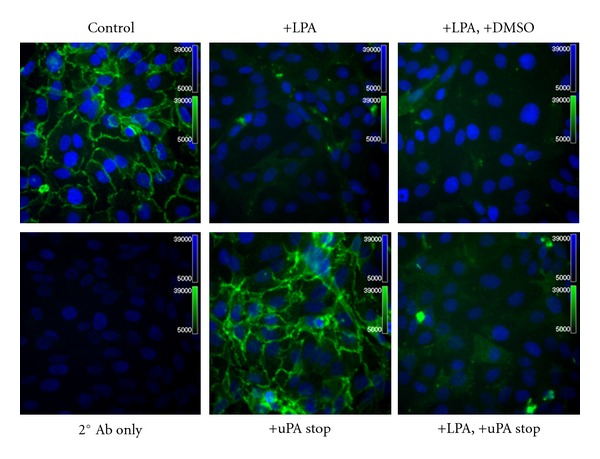
Inhibition of uPA activity does not prevent LPA-induced E-cadherin junction disruption. OVCA429 cells were treated with or without LPA (30 *μ*M), as indicated, for 18 hours in the presence or absence of the uPA inhibitor designated uPA Stop (2.5 *μ*M), as indicated and processed for immunofluorescence staining for E-cadherin using anti-E-cadherin ectodomain antibody (1 : 300) and Alexa Fluor 488-conjugated secondary antibody (1 : 500; green). Blue-DAPI-stained nuclei.

Primary EOC cells express abundant E-cadherin; however, E-cadherin staining in metastatic lesions is less prevalent [2, 3]. To evaluate a potential posttranslational mechanism for control of E-cadherin function, the effect of LPA on E-cadherin junctional integrity was evaluated. Treatment of OVCA 429, OVCA433, or OVCAR3 cells with LPA (20 *μ*M) resulted in a loss of junctional E-cadherin staining (Figure 1(a)). Detectable loss of E-cadherin staining was present as early as 2 hours (not shown) and was correlated with LPA concentration (Figure 1(b)). LPA levels as low as 1 *μ*M induced significant loss of junctional E-cadherin (Figures 1(b) and 1(c)). Although it was previously demonstrated that inhibition of the activity of urinary type plasminogen activator (uPA) could block LPA-induced E-cadherin junction loss [30], incubation of EOC cells with LPA in the presence of the inhibitor uPA-Stop did not result in maintenance of junctional integrity (Figure 2).

**Figure 3:**
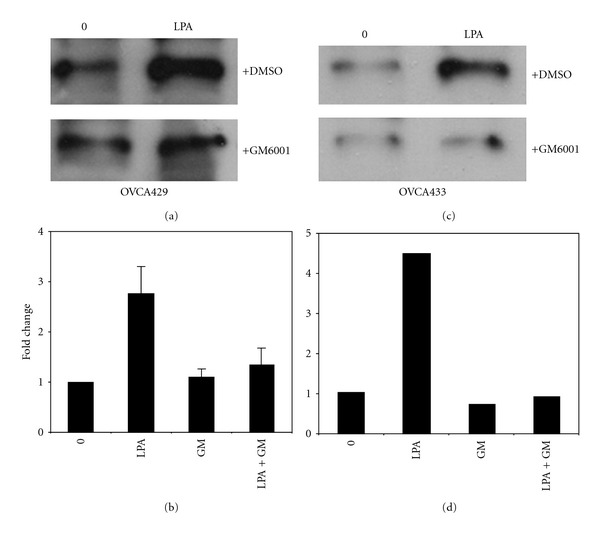
LPA induces MMP-dependent E-cadherin ectodomain shedding. (a, b) OVCA429 or (c, d) OVCA433 cells were treated with LPA (30 *μ*M) in the presence or absence of the broad-spectrum MMP inhibitor GM6001 (25 *μ*M) as indicated for 24 hours. Conditioned media were subjected to immunoprecipitation with anti-E-cadherin antibodies, and precipitates were western blotted with a second E-cadherin antibody (Zymed, 1 : 1,000) followed by peroxidase-conjugated secondary antibody (1 : 5,000) and enhanced chemiluminescence detection. Panels (b, d) show densitometric quantitation of replicate western blots.

**Figure 4:**
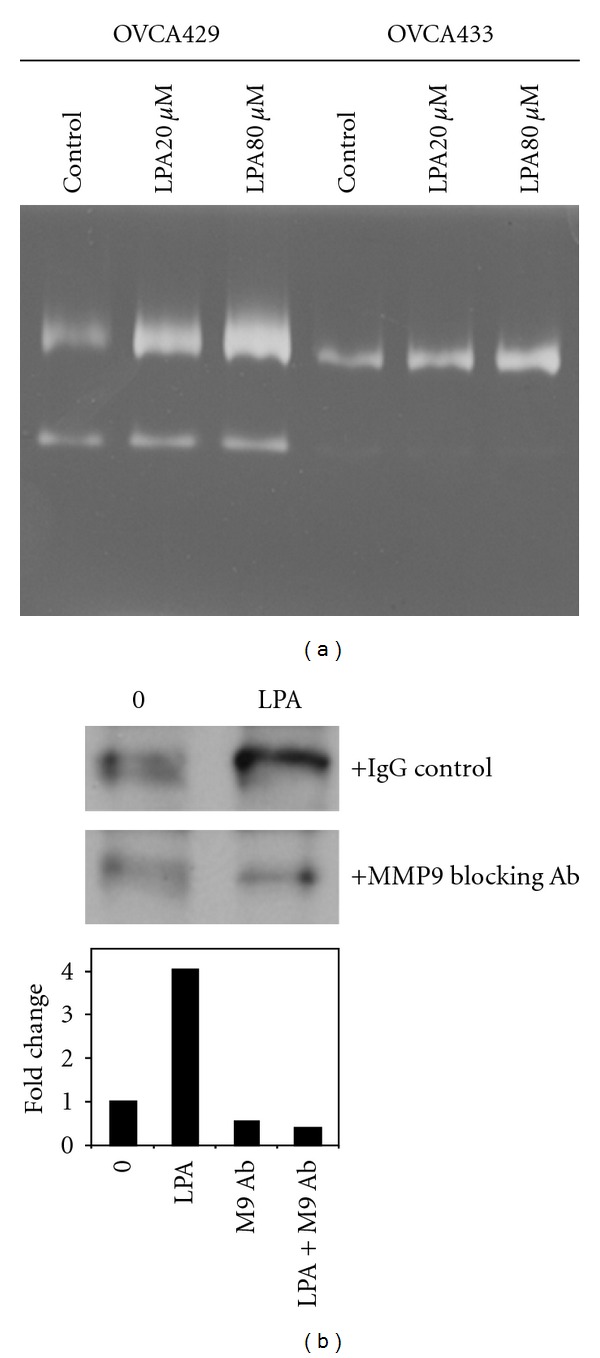
LPA induces MMP-9-dependent E-cadherin ectodomain shedding. (a) OVCA429 and OVCA433 cells were treated with LPA (0, 20, or 80 *μ*M, as indicated) and conditioned media evaluated for MMP expression by gelatin zymography. (b) Cells were treated with LPA (20 *μ*M) in the presence or absence of anti-MMP-9 function blocking antibody (10 *μ*g/mL). Conditioned media were subjected to immunoprecipitation with anti-E-cadherin antibodies, and precipitates were western blotted with a second E-cadherin antibody (Zymed, 1 : 1,000) followed by peroxidase-conjugated secondary antibody (1 : 5,000) and enhanced chemiluminescence detection. The lower panel shows densitometric quantitation of replicate western blots.

**Figure 5:**
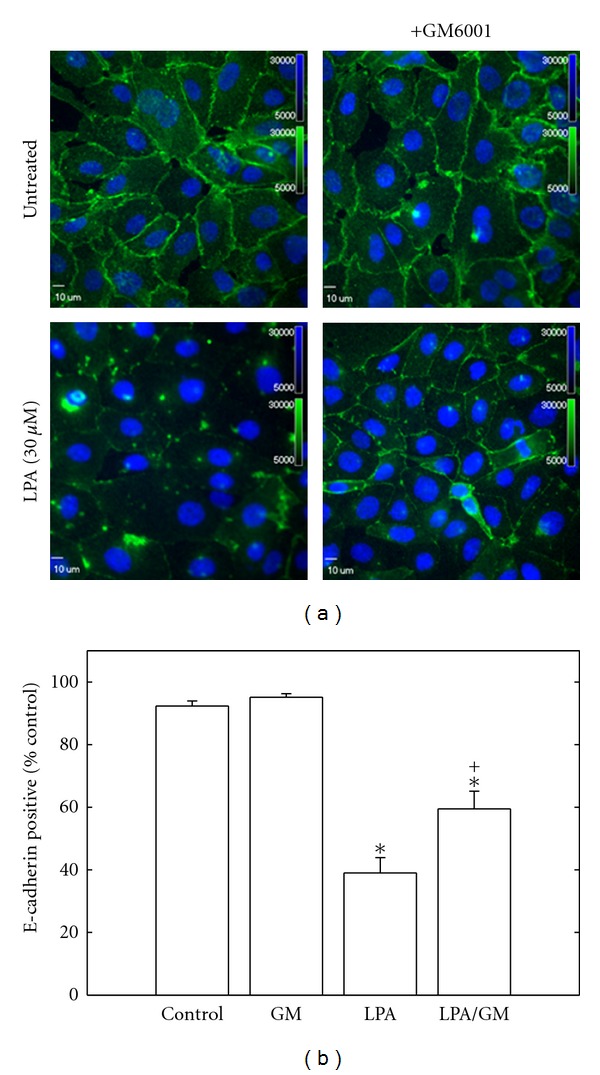
Blocking MMP activity inhibits LPA-induced junction loss. (a) OVCA429 cells were treated with LPA (30 *μ*M) for 24 hours in the presence and absence of the broad-spectrum MMP inhibitor GM6001 (25 *μ*M) as indicated and processed for E-cadherin immunofluorescence (green). (b) E-cadherin positive cells were quantified by scoring the number of cells with two remaining borders in a minimum of 12 fields per treatment. (*P < .0005 relative to control; ^+^ *P* = .007 relative to LPA).

**Figure 6:**
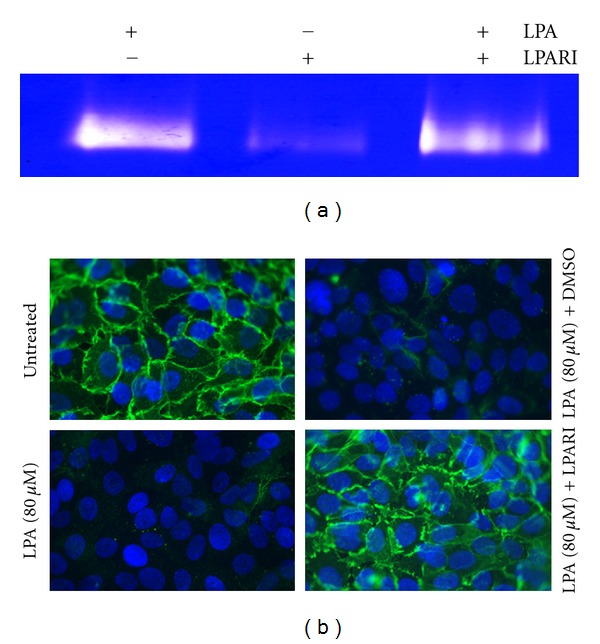
Inhibition of LPA receptor signaling blocks LPA-induced E-cadherin junction loss. OVCA429 cells were treated with LPA (80 *μ*M) for 24 hours in the presence or absence of LPARI (20 *μ*M) or DMSO (vehicle control), as indicated. (a) Conditioned media were examined by gelatin zymography. (b) Cells were processed for E-cadherin immunofluorescence using anti-E-cadherin ectodomain antibody (1 : 300) and Alexa Fluor 488-conjugated secondary antibody (1 : 500; green). Blue-DAPI-stained nuclei.

LPA increases MMP-9 mRNA expression in breast adenocarcinoma cells [31], and we have recently demonstrated that MMP-9 catalyzes E-cadherin ectodomain shedding in EOC cells [24, 28]. To evaluate whether the observed decrease in E-cadherin junctional staining may result from MMP-9-catalyzed E-cadherin ectodomain shedding, cells were incubated with LPA overnight, and conditioned media were subjected to immunoprecipitation using E-cadherin ectodomain-specific antibodies followed by Western blotting. Significantly increased shedding of the E-cadherin ectodomain was observed following LPA treatment (Figures 3(a)–3(d)). To assess the potential involvement of MMP(s) in E-cadherin ectodomain shedding, cells were treated with LPA in the presence of the broad-spectrum MMP inhibitor GM6001 (25 *μ*M). Incubation with GM6001 significantly reduced E-cadherin ectodomain shedding, implicating LPA-regulated MMP activity in this process (Figures 3(a)–3(d)). To identify whether MMP-9 may participate in LPA-induced E-cadherin processing, cells were treated with LPA (20 *μ*M and 80 *μ*M) for 24 hours and the resulting conditioned media examined via gelatin zymography. An LPA dose-dependent increase in MMP-9 expression was observed in both OVCA429 and OVCA433 cells (Figure 4(a)). To confirm the involvement of MMP-9, cells were treated with LPA in the presence of an anticatalytic MMP-9 function blocking antibody. Inhibition of extracellular MMP-9 activity blocked LPA-induced loss of cell surface E-cadherin (Figure 4(b)). This was confirmed by immunofluorescence microscopy, wherein treatment of cells with LPA in the presence of GM6001 resulted in a significant restoration of junctional integrity (Figure 5(a) and 5(b)). Further, inhibition of LPA signaling using the LPA receptor inhibitor LPARI (Ki16425; *K*_*i*_ 0.3–6.5 *μ*M for LPA_1–3_; working concentration 40 *μ*M) [27] reduced LPA-induced MMP-9 expression and blocked LPA-mediated junction dissolution (Figure 6).

**Figure 7:**
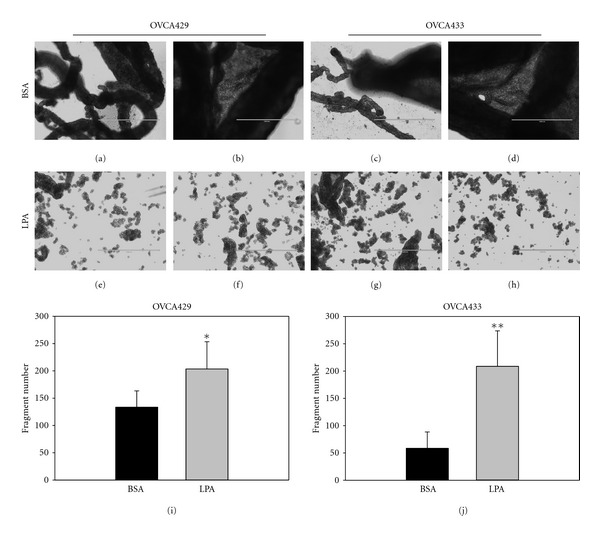
LPA treatment disrupts epithelial cohesion. Confluent layers of (a) OVCA429 and (b) OVCA433 cells were treated with LPA (40 *μ*M) for 24 hours. Cells were then detached from substratum as cell-cell adherent sheets using dispase (1 mg/mL). Cohesive epithelial sheets were transferred to tubes then subjected to 50 rotations on a bench-top rocker returned to a 60 mm culture dish, and total fragment number was enumerated. Top panels show (a, b) OVCA429 and (c, d) OVCA433 control cells (BSA treated) as intact epithelial sheets and cohesive multicellular strands. LPA treatment of (e, f) OVCA429 and (g, h) OVCA433 cells decreases epithelial cohesion, as evidenced by increased fragment number enumerated in (I, J). *P < .02; **P < .002. Scale bar 1000 *μ*m.

To assess the potential functional consequence of LPA-mediated loss of E-cadherin surface expression, epithelial integrity was examined using a dispase-based cohesion assay [30]. Confluent OVCA429 or OVCA433 cell monolayers were treated with or without LPA for 24 hours and were then detached from the culture dish as intact monolayers or cell sheets by dispase treatment, prior to application of rotational force to compromise monolayer integrity. In response to LPA treatment, significantly greater fragmentation of the cell sheet was observed compared with controls (Figure 7(a) and 7(b)) in both OVCA429 (37.4% increase, *n* = 6, *P* = 0.0203) and OVCA433 (70% increase, *n* = 6, *P* = 0.0015). This loss of epithelial cohesion highlights the functional relevance of the observed loss of junctional E-cadherin.

## 4. Discussion

Lysophosphatic acid was originally identified as “ovarian cancer activating factor” (OCAF) in 1995 [7]. Since its initial discovery, LPA expression has been linked to metastatic success in EOC through a variety of mechanisms. In EOC, LPA signals through a diverse subfamily of G-protein-coupled receptors (LPA_1–3_), to affect a variety of biologic processes including expression of extracellular proteinases and growth factors, alteration of stress fibers and focal adhesion dynamics, and enhancement of motility and invasion [12, 13, 15–17, 19, 20].

Results from the current study demonstrate a loss of surface E-cadherin expression in response to LPA in a dose-dependent manner. Concomitant LPA-induced MMP-9 expression results in MMP-9-catalyzed E-cadherin ectodomain shedding [24–28], while incubation with the broad spectrum MMP inhibitor GM6001 or the LPA receptor inhibitor Ki16425 reduces shedding. As a consequence of compromised junctional integrity, epithelial cohesion is significantly reduced, as evidenced by enhanced epithelial sheet fragmentation. Loss of epithelial cohesion together with gain of mesenchymal features is thought to accompany mesothelial anchoring of EOC metastatic lesions [32, 33].

It was previously reported that the serine proteinase urinary type plasminogen activator (uPA) can promote E-cadherin ectodomain shedding following a 4-hour LPA treatment [30], whereas inhibition of uPA activity using a small molecule inhibitor did not block E-cadherin loss in the current study using 24-hour LPA treatment. These data suggest that uPA-dependent shedding may be an early proteolytic event, while E-cadherin shedding is sustained by subsequent expression of MMP-9. This hypothesis is supported by previous data showing additional biologic events that promote MMP-9-dependent E-cadherin ectodomain shedding. For example, early events in EOC intraperitoneal metastatic dissemination involve integrin-mediated attachment to the multivalent submesothelial collagen matrix [2, 3]. Multivalent integrin engagement, in turn, induces MMP-9-dependent E-cadherin ectodomain shedding, promoting a potential mechanism for enhanced dispersal of metastatic cells [28]. Similarly, epidermal-growth-factor- (EGF-) induced activation of EGF receptor also potentiates loss of surface-expressed E-cadherin. This loss of E-cadherin expression is rescued in the presence of a broad spectrum MMP inhibitor or by siRNA silencing of MMP-9 expression [24].

Although elevated levels of soluble E-cadherin (sE-cad) were identified in cancer patients nearly two decades ago [34], the physiologic and pathophysiologic relevance of this polypeptide fragment remains poorly understood. sE-cad has been detected in ovarian cancer patient serum, and evaluation of the prognostic and diagnostic value of sE-cad serum concentrations is ongoing [35, 36]. sEcad is also highly prevalent in ascites fluid of women with EOC, reaching concentrations of over 12 *μ*g/mL, and addition of sEcad to EOC cells induces characteristics of epithelial-mesenchymal transition including junction disruption and morphologic alteration to a migratory phenotype [28]. In contrast to other tumors wherein shed sEcad is released into circulation, intraperitoneally localized primary and metastatic ovarian tumors maintain direct contact with sEcad-rich ascites. It is interesting to speculate that loss of E-cadherin during late metastatic progression facilitates epithelial-to-mesenchymal transition and confers a phenotype necessary for submesothelial invasive anchoring to promote intraperitoneal dissemination. LPA, therefore, represents a potent regulator of key events in EOC metastasis by enhancing MMP-9-dependent E-cadherin ectodomain shedding and promoting motility and invasion.
